# A knowledge-based framework for the discovery of cancer-predisposing variants using large-scale sequencing breast cancer data

**DOI:** 10.1186/s13058-017-0854-1

**Published:** 2017-05-31

**Authors:** Giorgio E. M. Melloni, Luca Mazzarella, Loris Bernard, Margherita Bodini, Anna Russo, Lucilla Luzi, Pier Giuseppe Pelicci, Laura Riva

**Affiliations:** 1 Center for Genomic Science of IIT@SEMM, Fondazione Istituto Italiano di Tecnologia, Via Adamello 16, Milan, Italy; 2 Department of Experimental Oncology, European Institute of Oncology, Via Adamello 16, Milan, Italy; 3 Division of New Drug Development, European Institute of Oncology, Via Ripamonti 435, Milan, Italy; 4 Clinical Genomics Lab, European Institute of Oncology, via Ripamonti 435, Milano, Italy; 5 Department of Oncology and Hemato-oncology, University of Milan, via Festa del Perdono 7, Milan, Italy

**Keywords:** Breast cancer, Predisposition, Germline mutations, Somatic mutations

## Abstract

**Background:**

The landscape of cancer-predisposing genes has been extensively investigated in the last 30 years with various methodologies ranging from candidate gene to genome-wide association studies. However, sequencing data are still poorly exploited in cancer predisposition studies due to the lack of statistical power when comparing millions of variants at once.

**Method:**

To overcome these power limitations, we propose a knowledge-based framework founded on the characteristics of known cancer-predisposing variants and genes. Under our framework, we took advantage of a combination of previously generated datasets of sequencing experiments to identify novel breast cancer-predisposing variants, comparing the normal genomes of 673 breast cancer patients of European origin against 27,173 controls matched by ethnicity.

**Results:**

We detected several expected variants on known breast cancer-predisposing genes, like *BRCA1* and *BRCA2*, and 11 variants on genes associated with other cancer types, like *RET* and *AKT1*. Furthermore, we detected 183 variants that overlap with somatic mutations in cancer and 41 variants associated with 38 possible loss-of-function genes, including *PIK3CB* and *KMT2C*. Finally, we found a set of 19 variants that are potentially pathogenic, negatively correlate with age at onset, and have never been associated with breast cancer.

**Conclusions:**

In this study, we demonstrate the usefulness of a genomic-driven approach nested in a classic case-control study to prioritize cancer-predisposing variants. In addition, we provide a resource containing variants that may affect susceptibility to breast cancer.

**Electronic supplementary material:**

The online version of this article (doi:10.1186/s13058-017-0854-1) contains supplementary material, which is available to authorized users.

## Background

Breast cancer is one of the most common cancers with more than 1,300,000 cases and 450,000 deaths per year worldwide [1]. It is caused, as any other tumor, by the accumulation of somatic mutations over time. Somatic mutations arise spontaneously in somatic cells and they are passed on to all descendants of these cells. The probability of acquiring mutations that can lead to breast cancer is sometimes increased by pre-existent germline variants that predispose to cancer or cause cancer-related syndromes. Germline variants are present in all the cells of a person and they can be inherited and passed on to the next generation. It is estimated that approximately 5–10% of women have germline mutations and polymorphisms that lead to hereditary predisposition to breast cancer [2]. Although specific mutations in *BRCA1* and *BRCA2* are known to be responsible for inherited susceptibility to breast cancer in families with early-onset disease [3], *BRCA1/2* mutation carriers account for just 20% of the enhanced risk in first-degree relatives [3]. Mutations in other genes, such as *PALB2*, *PTEN* and *TP53*, have been also associated with increased risk of breast cancer. Nevertheless, many familiar breast cancers (approximately 50%) are still unexplained at the genetic level and many predisposing variants are yet to be found [4].

Beside the use of linkage analysis, which requires families with a penetrant phenotype, the discovery of the majority of well-known cancer-predisposing genes (CPGs) has been through the analysis of candidate genes [5]. To shed light on the remaining hidden heritability in breast cancer, genome-wide association studies (GWAS) have been extensively carried out [6]. A large meta-analysis and integration of multiple GWAS carried out by the Collaborative Oncological Gene-environment Study (COGS) consortium led to the identification of dozens of susceptibility loci [4, 7]. However, GWAS suffer from a number of well-recognized limitations. First, they can only suggest the *regions* where the pathogenic variants might actually reside, but not their identity. Second, they rely on single nucleotide polymorphisms (SNP, by definition occurring in >1% of the population) and thus are poorly suited to identify rare variants. Whole exome sequencing (WES) and whole genome sequencing (WGS) can theoretically overcome most of these limitations. WES/WGS-based studies to investigate breast cancer-associated risk variants have not been carried out to date, since the size imposed by the millions of variants to be tested simultaneously is technically unreachable [8]. However, germline sequencing is routinely performed in projects aimed at identifying somatic tumor variants. Indeed, looking at matched germline DNA in a consecutive series of tumors sequenced to find somatic mutations, it could be demonstrated that disease susceptibility due to rare variants in sporadic cancers is much more common than previously anticipated [9].

Large collections of WES/WGS of both tumor patients and healthy subjects are available through multicentric efforts like The Cancer Genome Atlas (TCGA) [10] and the Exome Aggregation Consortium (ExAC) [11] and can be leveraged to identify putative risk variants. For reasons highlighted above, a straightforward case-control comparison on allele frequencies would be underpowered. Thus, we planned a computational framework based on our knowledge of the characteristics of known cancer-predisposing genes and variants. In particular, we took advantage of the characteristics of somatic driver genes (like their gain or loss-of-function) to reproduce a candidate gene analysis. Cancer is, in fact, a unique example of disease causation and disease predisposition, being strongly linked to clear definitions of gain-of-function/oncogenes and loss-of-function/tumor suppressor genes [5]. Using these cancer unique characteristics, we have been able to identify a set of variants and genes that may affect susceptibility to breast cancer.

## Method

### Study design

**Fig. 1:**
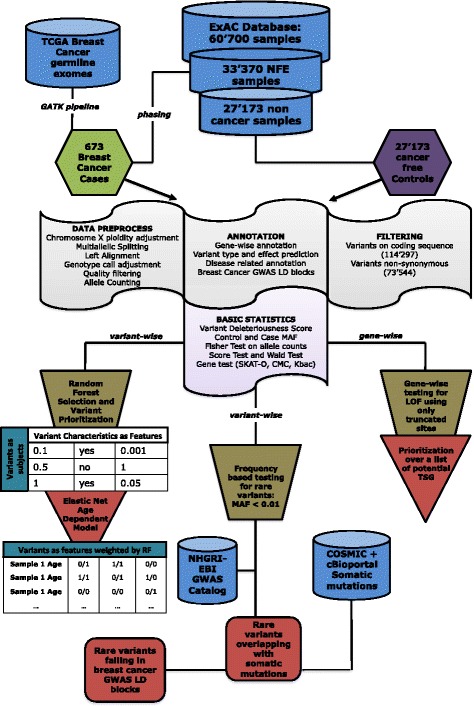
Workflow scheme for the whole analysis. *Blue cylinders* represent the data (obtained from available databases or processed during the analysis); *hexagons* are the analyzed datasets of cases and controls; *red squares* and *triangles* represent analysis and output. *Flag shapes* represent post-process annotation and statistical testing; *brown trapezoids* represent the three main analysis branches presented in this paper. *NFE* non-Finnish European

We designed this study as a classic case-control study, with emphasis on variants rather than entire genes. In particular, we took advantage of several databases for the annotation of the variants, to produce a hypothesis-based framework that could preselect valid candidates and apply statistical tests afterward. Within this framework, we studied the normal genomes coming from 673 breast cancer patients of European origin from the TCGA against over 27,000 control genotypes, unselected for cancer phenotype, from the ExAC database with matched ethnicity (Fig. 1).

### Study data

#### Case dataset

We downloaded from the TCGA the original Binary Alignment/Map (BAM) files of the normal sample for all the 695 women and men of Caucasian origin diagnosed with breast cancer. We then used 673 (7 men and 666 women) of the 695 samples, considering only WES data and blood-derived normal samples. We analyzed the BAM files, following the exact same Genome Analysis Toolkit (GATK) pipeline and the same level of sensitivity used for the control dataset (see Additional file 1: Data preprocess). We retrieved from the TCGA open access database the available clinical information for these patients, including age and sex, estrogen receptor (ER) status and molecular subtypes.

#### Control dataset

We used the aggregated results from the ExAC database as control population. This resource aggregates more than 60,000 samples with germline genotype data, of which 33,370 are classified as of European origin. The original data source is both from population studies (1000 Genome Project, HapMap, Exome Sequencing Consortium) and from disease-related studies (including part of the TCGA). To avoid any overlap with tumor samples, we filtered our data against cancer samples, remaining with a total of 53,105 samples, of which 27,173 were of Caucasian origin.

#### Annotation data

To perform our analysis we took advantage of several resources and custom-made datasets to annotate and filter variants. In particular we used:

1. Gene, protein change, type of variants and predicted phenotypic effect from nine different tools using ANNOVAR annotations [12]. The phenotypic effect was summarized as a deleteriousness score (DS), calculated as the proportion of tools calling a particular variant as damaging or probably damaging [13].
2. Presence of the variants in “target genes”, a manually curated list of 758 genes known to be cancer predisposing, somatic cancer drivers or belonging to DNA repair pathways (see Additional file 2: Table S1 and S2 and Additional file 3)
3. Annotation for the possible linkage disequilibrium between our variants and cancer-associated SNPs from the Human Genome Research Institute’s Catalog of Published genome-wide association studies (NHGRI-EBI GWAS Catalog) [14] (Additional file 2: Table S3 and S4). Each SNP define a linkage disequilibrium (LD) region, called LD block.
4. Annotations regarding the presence of the variants in cancer-specific databases Clinic Interpretation of Variants in Cancer (CiViC) [15], Database of Curated Mutations (DoCM) [16], Catalogue of Somatic Mutations in Cancer (COSMIC) [17], cBioPortal [18]) and in databases of pathogenic variants (HumSavar [19], ClinVar [20]). Cancer-related pathogenic variants are used in particular to define a set of prototype in Results – age-dependent polygenic model (Additional file 2: Table S5).

Further details can be found in Additional file 1: Annotation data.

### Statistical analyses

Statistical power is a critical issue in genome-wide case-control studies. In particular, exome data are even more underpowered than GWAS, since potentially millions of variants can be tested at a time. The initial call from all the 673 samples included millions of variants that were filtered to keep only coding and non-synonymous events. Since we did not perform any imputation and we applied a strict quality filter after the raw calls, retaining only exonic variants was the best way to maximize coverage in a dataset composed for the large majority by exome sequencing data (including all cases). We thus performed the following three analyses (Fig. 1):

1. *Hypothesis-driven analysis of rare variants in target cancer genes*. We devised the following scheme to prioritize candidate cancer-predisposing variants in target genes:
*Identification of variants in known breast cancer-predisposing genes*
*Identification of variants in CPGs with no known association with breast cancer*
*Identification of somatically mutated germline variants*. A germline variant able to favor carcinogenesis may have a higher chance of being also somatically mutated in cancer genomes [5]. We called these variants (“somatically mutated germline variants”, SMGVs). To identify SMGVs, we composed a large set of *somatic* variants from WGS and WES studies, combining the COSMIC and cBioPortal databases, and checked for possible matches between germline variants and somatic mutations (see Additional file 1: Annotation data).
2. *Tumor suppressor-like analysis*. All known breast CPGs, and at least 90% of all CPGs across cancer types, show their oncogenic potential through loss-of-function mutations. In the same way, tumor suppressor genes at the somatic level are generally hit by truncating mutations (nonsense or frameshift insertions or deletions) that disrupt the original function of the gene. We therefore selected truncating variations in our dataset and checked for any positive imbalance between the minor allele frequency (MAF) in the cases and in the controls gene-wise (see Additional file 1: Annotation-based analysis).
3. *Age-dependent polygenic modeling*. In this analysis we put no filter on frequency by running a completely unbiased regression analysis on all non-synonymous variants over age at onset. As explained in the section Additional file 1: Age-dependent polygenic model, we implemented a double-step machine-learning approach composed by (1) a tree-based supervised classification with variants as subjects (dimensionality reduction step via random forest), and (2) a penalized linear model regressing the age to the cases’ genotypes, so that the variants become now covariates (feature selection step via elastic net).

Detailed information about these three analyses can be found in Additional file 1: Statistical analyses.

## Results

### Hypothesis-driven analysis of rare variants in target cancer genes

#### Variants in known breast cancer-predisposing genes

We first asked whether known breast cancer-predisposing variants were present in our dataset. We collected a list of 15 known breast cancer susceptibility genes from the literature (Table 1) [5, 21, 22] and checked for variants in the TCGA dataset, considering both known pathogenic and truncating variants (Table 1). We decided to take into account also rare truncating variants, since they are generally considered de facto pathogenic when the gene exerts its oncogenic function via loss-of-function. This is the case for all the known predisposing genes in breast cancer and, in general, for the large majority of CPGs.

**Table 1:** Breast cancer-predisposing genes and variants found in our case dataset

Gene	Somatic driver gene	Total number of variants	Number of pathogenic variants	Number of truncating variants	Number of highly damaging mutations
*ATM*	X	21			5
*BRCA1*	X	18	2		3
*BRCA2*	X	21	5		2
*BRIP1*		5	1		
*CDH1*	X	3	1		
*CHEK2*	X	6	2	1	
*MRE11A*		4			1
*NBN*		5	1	2	
*PALB2*		1			
*PRKAR1A*					
*PTEN*	X				
*RAD50*		5			
*RAD51C*		3		1	
*STK11*	X	3			
*TP53*	X	4			1

The second column reports if the gene is also considered to be a somatic driver gene. The next three columns report the total number of non-synonymous variants, the number of variants considered being pathogenic, and the number of rare truncating variants (control minor allele frequency below 1%) not already included in the list of pathogenic variants. The last column shows instead all the missense variants that are not considered to be pathogenic but have a very high deleteriousness score (8/9 tools for predicting functional damage report the variant as damaging). As pathogenic reference we used the ClinVar and Humsavar databases

We obtained 16 different mutations that cover 36 of our 673 cases (approximately 5%). The frequency of the identified variants in the breast cancer dataset is compatible with a sample of sporadic cases, especially given the fact that many potential pathogenic variations are still not reported in databases like ClinVar [20]. We found no variation for both *PTEN* and *PRKAR1A* (Table 1) but it is rare to find mutations on these genes. The cancer syndromes linked to them (Cowden syndrome and Carney complex) are in fact extremely infrequent in the population: the former has an incidence of one in 200,000 individuals [23], the latter a total prevalence of few hundred reported cases [24].

#### Variants in CPGs with no known association with breast cancer

Many CPGs are associated with complex tumor syndromes or have multiple tumor associations in which more than one tumor type can arise [5]. Known examples are the aforementioned *BRCA1* and *BRCA2* that are linked to both breast and ovarian cancers [25], or the more recent discovery of *PALB2*, linked to breast and pancreatic tumors [26, 27]. We therefore looked for any variant connected to additional cancers or cancer syndrome genes and, on 11 genes, we found 11 different rare variants that showed a higher MAF in the cases than in the controls (Table 2). The reference list of cancer-related variants was derived from Humsavar, ClinVar, DoCM and CiViC (see Additional file 1: Annotation data). Some of these variants are extremely rare, found in one patient over 673 and therefore they would fail any statistical test trying to assess their enrichment in cancer. Nevertheless, our hypothesis-driven approach allowed us to identify them as candidates among thousands of rare variations.

**Table 2:** List of rare cancer-related pathogenic variants [control minor allele frequency (MAF) below 1%]

Gene – variant	Control MAF	Case MAF	log2 MAF ratio	Summary of ClinVar and Humsavar annotations
*COL7A1 - R1538C - (3,48619779,G,A)*	0.002%	0.07%	5.35	Malignant melanoma
*AKT1 - E17K - (14,105246551,C,T)*	Novel	0.08%	4.47	Colon, ovary and breast cancer
FANCC - R185* - (9,97912338,G,A)	0.006%	0.07%	3.76	Fanconi anemia
*MSH6 - T955fs - (2,48030639,-,C)*	0.213%	2.61%	3.62	Lynch syndrome
*ELAC2 - R741H - (17,12896274,C,T)*	0.072%	0.23%	1.66	Prostate cancer
*RET - Y791F - (10,43613908,A,T)*	0.244%	0.69%	1.50	MEN2A syndrome/thyroid carcinoma
*FLCN - R239C - (17,17125879,G,A)*	0.033%	0.08%	1.20	Renal cell carcinoma
*PKHD1 - T36M - (6,51947999,G,A)*	0.075%	0.15%	0.98	Renal cancer
*GALNT12 - D303N - (9,101594229,G,A)*	0.185%	0.30%	0.72	Colorectal cancer
*PRF1 - N252S - (10,72358722,T,C)*	0.501%	0.82%	0.72	Non-Hodgkin lymphoma
*SDHD - G12S - (11,111957665,G,A)*	0.992%	1.04%	0.07	Cowden disease 3

This list includes all those genes that are not breast cancer predisposing but are connected to other types of cancer or cancer syndromes.

*translation termination (stop) codon

Among the genes, we found *COL7A1*, a collagen gene linked to epidermolysis bullosa, a severe skin syndrome with elevated lifetime risk of melanoma [28]. We also detected a variant on *RET*, a gene connected to MEN2A syndrome that confers an extremely high penetrant risk of thyroid cancer [29]. To our knowledge, *RET* has been connected to breast cancer only through deregulation in its expression levels [30]. Evidence of a connection to another thyroid cancer-related syndrome (MEN1) was recently demonstrated in breast cancer [31], but a possible link to MEN2A is novel and, if confirmed, would represent an unusual case of a gain-of-function mutation linked to breast cancer risk. Interestingly, we identified three truncating or frameshift alterations on *FANCC*, *FLCN*, and *MSH6*, three loss-of-function genes associated, respectively, to Fanconi anemia (like *PALB2, BRCA1,* and *RAD51C* reported in Table 1) [32], renal cell carcinoma [33], and Lynch syndrome [34], with no previous direct connections to breast cancer. Lastly, we discovered *AKT1* E17K, a variant linked to many cancer types, including breast cancer, at the somatic level. It is reported in databases such as ClinVar or OMIM [35] (that are generally focused on hereditary genetic traits) because it is considered a high-frequency somatic driver mutation [36]. This gene was also linked to a minority of Cowden syndrome cases along with *PIK3CA* since it belongs to the same pathway as *PTEN,* whose mutations are causative of 85% of the cases [23]. This variant is particularly relevant because it represents both an example of a gain-of-function mutation in a breast cancer frequently somatically mutated oncogene and a risk-associated germline variant in our dataset.

#### Co-occurrence of known cancer-predisposing variants

**Fig. 2:**
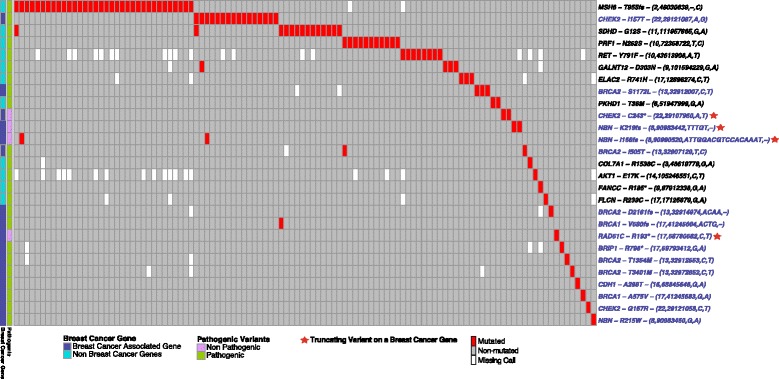
Distribution of pathogenic and truncating variants on breast cancer genes in our case dataset of 673 breast cancer patients. Oncoprint plot showing three classes of high confidence breast cancer-predisposing genes (*rows*); each column represents one of the samples with at least one of these mutations. Variants on known breast cancer-predisposing genes are indicated in *blue* (complete list in Table 1). A *star* indicates a variant that is a truncation but is not reported as pathogenic in the ClinVar or Humsavar databases. Pathogenic variants which affect genes related to cancer or cancer syndromes that are not linked to breast cancer are indicated in *black* and include genes like *RET* (thyroid cancer) or *APC* (colon cancer)

To summarize our findings, we drew a co-occurrence heatmap of all the aforementioned variants in our dataset (Fig. 2). The sum of all the cases with at least one of these mutations is 110 and approximately covered 16% of our dataset and included 11 non-breast-related pathogenic variants, 12 pathogenic breast-related variants, and four truncating variants on breast CPGs. However, co-occurrent mutations were quite rare: only seven of the 110 samples had more than one variant. Furthermore, variant frequency in the dataset was extremely unbalanced: the top eight variants in Fig. 2 account for 13% of patients, while 19 variants cover the remaining 3%.

#### Somatically mutated germline variants

**Fig. 3:**
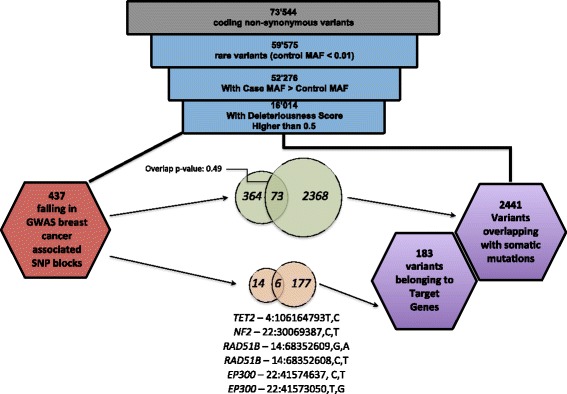
Analysis of rare variants. This flowchart represents the step-wise procedure in the central arm of Fig. 1 and is performed by filtering 73,544 non-synonymous coding variants down to 16,014 rare variants (MAF <1%), with a deleteriousness score over 0.5 and where the MAF in the cases is higher than in the controls. Rare variants are prioritized into two branches: on the *left*, variants falling in GWAS breast cancer linkage disequilibrium blocks (LD blocks); on the *right*, variants overlapping with cancer somatic mutations from COSMIC or cBioPortal (see Additional file 3: Table S6 and S7). For both datasets, overlaps are shown both at the initial level and after filtering for variants belonging to our list of 758 target genes (known cancer-predisposing genes, known somatic driver genes, and DNA repair genes). Six common (i.e., both overlapping with somatic mutations and falling into a GWAS LD block) variants on our target gene list, are reported at the bottom of this figure

We identified approximately 70,000 non-synonymous variants; of these we kept the rare variants which showed a higher prevalence in the cases respect to the controls (approximately 50,000). We then computed their deleteriousness score (DS), by adding up the individual scores obtained through the nine different methods evaluating the functional impact of the mutations, and retained the variants with a DS >0.5 (i.e., those defined as damaging in four or more methods, see Additional file 1: Annotation data) [13]. DS filtering yielded approximately 16,000 variants (Fig. 3). Unbiased inference of causality for such rare variants would require exceedingly large datasets. However, the results described above suggest that a simple comparison of variant allele frequencies in the TCGA cases using sufficiently large control datasets allows re-discovering known risk associations. Thus, it should be possible to transfer this approach to exploratory analyses aimed at identifying candidate loci to be validated externally. Accordingly, we further selected our variations, keeping only perfect matches with the somatic mutations in the COSMIC and cBioPortal databases and 440 variants were finally retained; of these, 183 belong to our list of manually curated target genes reported in Additional file 3: Table S6 (see Additional file 1: Annotation data). Among the 183 variants, we found 37 monomorphic alterations in the ExAC database that represented our control. The most relevant result of this analysis branch is probably the already mentioned variant E17K on *AKT1* (rs121434592). The *AKT1* gene is a known somatic driver kinase and this mutation was found in the cBioPortal database in 46 different samples from many different tumor types, including breast. E17K is also in the CIViC and DoCM database lists of curated somatic driver mutations [36]. Along with *ATM* R337C (rs138398778), this variant is in the list of cancer hotspots curated by Chang et al. [37], both variants representing a case of known somatic driver mutation that can be considered a cancer-predisposing variant. In addition, we found other germline variants present in more than two samples in COSMIC or cBioPortal on the following genes: *HNF1A, FGFR3*, and *ASXL1*. Interestingly, these genes are included in our list of CPGs or somatic driver genes, and none of them has been connected to breast cancer predisposition before.

We also developed a way to annotate if a variant falls close to at least one of 130 manually selected breast cancer-associated SNPs from the NHGRI-GWAS catalog (see Additional file 1: Annotation data), which are reported as *GWAS blocks* in Additional file 2: Table S3–4 [14]. A variant in proximity of one these SNPs can be considered in linkage disequilibrium with the SNP itself. We identified 436 variants within the GWAS blocks associated with breast cancer (Additional file 3: Table S7). Using STRING [38], we found that the genes containing these variants had more interactions among themselves than what would be expected from a random set of genes of the same size (*p* value: 0.000279). Interestingly, three genes in our list (*ZNF365*, *SGSM3*, and *LSP1*) were significantly annotated (using Webgestalt [39] and Disgenet [40]) with the “category mammographic density”, that is a strong risk factor for breast cancer [41]. Among the list of SMGVs, only 73 out of 436 fell in such GWAS regions (Fig. 3 and Additional file 3: Table S7). The overlap of these two groups is apparently random as it is not significantly different from a bootstrap of random overlaps (*p* value of permutation Z test = 0.19). This result highlights two important aspects. First, the lack of enrichment in somatic mutations in GWAS-associated regions confirms the results of Machiela et al. [42], which show no correlation between regions around cancer-associated SNPs and enrichment in somatic mutations. Second, while GWAS are designed to work on common variants, somatic mutations are usually rare. Thus, these two types of analysis represent two different layers of hereditability. After subsetting for our list of target genes, only six variants in four genes ended up being SMGVs and fell in the GWAS regions. These variants form a list of highly valuable candidates (reported at the bottom of Fig. 3), as theorized by one of the COGS flagship papers [7]. In particular, *RAD51B* is a known breast cancer-associated gene [43]. *TET2*, another variant discovered in our dataset, is only approximately 80 kb away from the COGS SNP rs9790517. Notably, *TET2* has been already associated with breast cancer at the RNA level [44] and it is considered a known somatic driver in leukemia and melanoma [45]. Another COGS variant (rs132390 on *EMID1*) is in a low recombination region together with the *NF2* R335C variation. *NF2* has been associated with hereditary neurofibromatosis syndrome 2 and it is mutated at both germinal and somatic levels in breast cancer [46]. The same COGS SNP was found in LD with *CHEK2,* a known breast cancer-associated gene [47]. Although our HapMap data do not support this linkage disequilibrium, we found a variant on *CHEK2* (rs201206424) at approximately the same distance as the *NF2* variant described above (approximately 400 kb) [7]. This *CHEK2* variant was also found as somatically mutated in breast cancer in our database. Finally, we found two different alterations on *EP300* in LD with the COGS SNP rs6001930. *EP300* has a well-established role as a tumor suppressor gene but has been poorly investigated as a breast cancer-predisposing gene [48].

#### Tumor suppressor-like analysis

The large majority of CPGs exerts their function via recessive loss-of-function variants [5]. We selected from our dataset all the truncating variants occurring below 1% in the control population: in total 2372 different truncating events on 1865 different genes. On this reduced dataset, we looked for any imbalance between control and case frequency in any of the truncating spots with a gene-wise testing procedure (see section Additional file 1: Tumor suppressor-like analysis). After testing and correcting for false discovery rate (FDR) [49], we looked for potential tumor suppressor-like genes in our list of 758 target genes. Only 90 genes had at least one truncating variant with a frequency in the control cohort below 0.01; of these, 38 passed the *p* value threshold (Additional file 3: Table S8). As a proof of concept, known breast cancer-predisposing genes like *BRCA1*, *BRCA2*, and *CHEK2* were selected by our procedure. Other known breast cancer-predisposing genes, such as *TP53* or *PALB2*, were instead not found truncated in our dataset because they are too rare for our detection power in a non-familiar selected dataset (Table 1) [50]. Nevertheless, *TP53* has one missense variant included in the list of the 183 variants overlapping with somatic mutations, and this particular variant was never reported as pathogenic before (rs138729528). Among the 41 significant LOF candidates, *FGFR3*, *PIK3CB*, *HNF1A*, and *KMTC2* were also highlighted as somatically mutated by the previous analysis, but, in this case, we were able to add a possible loss-of-function role. Interestingly, the genetic ablation of the protein encoded by *PIK3CB* was described to increase ductal branching and tumorigenesis and could lead to mammary gland hyperplasia in transgenic models of breast cancer [51]. In addition, the landscape of the somatic mutations of *PIK3CB and FGFR3* could be an indication that their inactivation might promote cancer progression. In fact, *PIK3CB* and *FGFR3* had a higher presence of somatic truncating events (26.1% and 15.7% respectively, as reported by cBioPortal), compared with the number of truncations that are present in a typical gain-of-function oncogene like *KRAS* or *PIK3CA* (approximately 1%). The majority of the genes in the tumor suppressor-like list harbors one to two different truncation points. *CRIPAK*, however, appears to be an exception with 27 different truncations in various points of the gene body. The abundance of frameshifts and nonsense alterations at various points of the protein can be partially explained by the fact that *CRIPAK* is an intronless gene containing multiple repetitions of a 31 bp sequence and, like other genes with this feature (e.g., *CDR1* or *AD7C-NTP*), tends to accumulate these variations for evolutionary reasons [52]. In fact, a recent publication on the impact of loss-of-function mutations on coding genes scored *CRIPAK* among the most tolerant genes (0.99 on a scale between 0, low tolerance, and 1, high tolerance) [53]. In addition, due to the repetitive nature of its coding sequence, it is easier to make alignment mistakes [54]. We think it is most likely a false positive result.

#### Age-dependent polygenic model

**Fig. 4:**
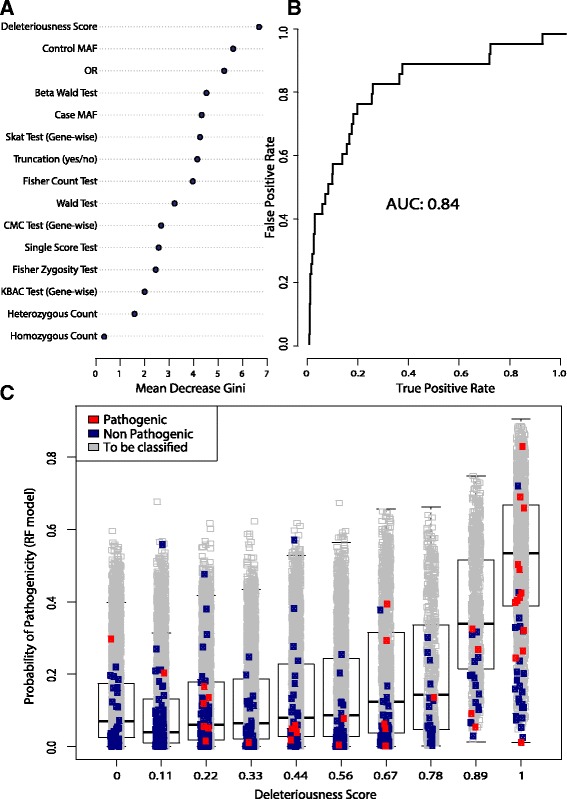
Polygenic age-dependent model breakdown. **a** Feature ranking of the random forest model according to the mean decrease of Gini index. At the *top*, the most important variable is the deleteriousness score. **b** ROC curve on random forest training model. An AUC of 0.84 is reached under the supervision of the training dataset formed by reported pathogenic and non-pathogenic variants according to the ClinVar and Humsavar databases. **c** Representation of the distribution of deleteriousness score among variants. The top predictor in our random forest model is shown without the influence of the other variants. Although it cannot represent the real tree scheme of the model alone, there is a clear positive trend between increased deleteriousness score (DS, X-axis) and the number of trees classifying a variable as pathogenic (RF score, Y-axis)

In the last part of our work, we moved from a pure case-control study to a more association-like study. Using the algorithm described in the Additional file 1: Age-dependent polygenic model, we determined the main characteristics of pathogenic and non-pathogenic variants, as shown in Fig. 4a, and we used these characteristics to classify the test set of unknown variants (all the remaining non-synonymous variants). The overall model on the training set reported a very low error in the classification process (out-of-bag error equal to 3.5%), with an area under the receiver operating characteristic (ROC) curve of 0.84 (see Fig. 4b). For example, as shown in Fig. 4c, the random forest model has the tendency of assigning high RF scores to the most deleterious variants, as a clear linear trend is visible between the DSs and the RF scores (see in Fig. 4c). The majority of the known pathogenic variants (the red dots) fall into the top two DS categories, compared to the non-pathogenic variations (the blue dots), which appear to fall in every category without a specific pattern. However, the DS is not sufficient to classify the training set properly, and only the integration of the other features results in a very low classification error (Fig. 4a, b).

After the first dimensionality reduction step, we retained 4045 variants entering the second step, and therefore reducing the risk of an inflated dimensionality (Additional file 1: Age-dependent polygenic model). We regressed the age at the initial pathological diagnosis to the genotype of our subjects in order to obtain a list of variants negatively associated with age at onset. The controls in this procedure are therefore not included. The final output in Table 3 shows 19 variants, ordered by the number of times a feature is retained with a negative beta in a model in at least 10% of the 100 models; 15 variants had a negative beta in more than 50% of the 100 models (see Additional file 1: Age-dependent polygenic model).

**Table 3:** Results from the polygenic age-dependent model

Variant	Approved name	Control MAF	Case MAF	Protein change	Mean beta elastic net	Negative beta percentage
*MRPL24 - 1,156708335,C,T*	Mitochondrial ribosomal protein L24	Novel	0.074%	W54^*^	−2.78	1.00
*CST4 - 20,23667825,-,C*	Cystatin S	0.0129%	0.300%	V81fs	−5.09	1.00
*PARD6A - 16,67696278,C,T*	Par-6 family cell polarity regulator alpha	0.0018%	0.078%	R256*	−1.86	1.00
*TRIOBP - 22,38121788,-,C*	TRIO and F-actin binding protein	0.0059%	0.471%	S1075fs	−3.64	1.00
*ZNF85 - 19,21132125,C,T*	Zinc finger protein 85	Novel	0.085%	R205*	−4.36	1.00
*FOXP4 - 6,41553185,A,G*	Forkhead box P4	0.0018%	0.091%	K147R	−8.04	1.00
*PKHD1 - 6,51890490,A,C*	Polycystic kidney and hepatic disease 1 (autosomal recessive)	Novel	0.075%	M1373R	−5.33	1.00
*SURF1 - 9,136218808,A,T*	Surfeit 1	Novel	0.081%	L179Q	−6.49	1.00
*HIST2H2AB - 1,149859084,TT…GT,-*	Histone cluster 2, H2ab	Novel	0.074%	T121fs	−3.59	0.97
*STIM2 - 4,27004586,G,A*	Stromal interaction molecule 2	Novel	0.081%	V281I	−1.65	0.97
*CPA3 - 3,148597632,C,T*	Carboxypeptidase A3 (mast cell)	Novel	0.074%	R178^*^	−5.47	0.94
*TMCO3 - 13,114188422,-,G*	Transmembrane and coiled-coil domains 3	0.0326%	0.742%	A469fs	−1.93	0.93
*SERPINF2 - 17,1649022,CCTG,-*	Serpin peptidase inhibitor, clade F	Novel	0.080%	A62fs	−1.74	0.84
*PYGL - 14,51383751,G,A*	Phosphorylase, glycogen, liver	0.0037%	0.149%	R276C	−0.08	0.71
*FNIP2 - 4,159790466,C,A*	Folliculin interacting protein 2	0.0016%	0.101%	S893^*^	−0.86	0.58
*CPPED1 - 16,12758817,G,A*	Calcineurin-like phosphoesterase domain containing 1	Novel	0.074%	R149^*^	−0.14	0.44
*OR52B4 - 11,4388943,G,A*	Olfactory receptor, family 52, subfamily B, member 4 (gene/pseudogene)	0.0018%	0.076%	R195^*^	4.81	0.09
*SCN10A - 3,38755496,G,A*	Sodium channel, voltage gated, type X alpha subunit	0.0037%	0.074%	R1155C	1.62	0.08
*ZNF683 - 1,26694960,G,A*	Zinc finger protein 683	Novel	0.089%	R35*	1.18	0.03

A double-step machine learning algorithm selects variant based on a series of pathogenic prototypes and then further selects them using a permutation-based multi-model regression over age at onset. Variants in this set are negatively associated with age, and are divided in three layers: at the top, variants negatively associated in at least 80% of the models and with an average beta less than −1.5; in the middle, variants retained in at least 40% of the models with poor average beta; at the bottom, variants found negatively associated only in a few models

*translation termination (stop) codon

We noticed several desirable features of the final set of variants. First, without imposing a filter on the control MAF, we selected for rare variants in the population, so that all our 19 variants have, in the control set, a MAF way below the 1% threshold and, in the case set, a MAF for the corresponding variants higher than in the controls. Furthermore, more than a half of these variants were not present in the ExAC dataset. Second, 13 of the 19 variants are classified as truncation events and all the other six missense events have a deleteriousness score higher than 0.8, thus judged as almost certainly damaging. Third, we noticed a double enrichment in variants also found as somatic variants, confirming the importance of evaluating somatic events overlapping with germline mutations.

Among the initial dataset of 73,354 variants, only approximately 13% of them were found as somatic events in COSMIC or cBioPortal. After the random forest procedure, this frequency had increased up to approximately 17% among the 4045 retained variants (*p* value of binomial test: 8.78e-11), and after the elastic net selection up to approximately 26% (five out of 19 variants were also found as somatic, although this is not significant due to the low number of variants).

None of the genes found using this procedure belongs to the list of target genes, none are found within low recombination regions of breast cancer-associated SNPs, and there are very few literature reports of a known involvement in cancer, thus making their selection a completely novel finding (Table 3). Excluding variants on *TMCO3*, *TRIOBP*, *PYGL*, and *CST4*, all the remaining 15 variants involved one single sample from our dataset; since they are so rare, a simple statistical approach would probably not detect them. As briefly mentioned before, this set of variants and genes are mostly not known to be in cancer, except for *PKHD1*, a gene involved in polycystic kidney disease and associated with a higher risk of renal cancer [55]. Another known pathogenic variant in *PKHD1* has also been mentioned in the first section of the results. Other genes with some evidence of cancer involvement among those reported in Table 3 include *STIM2*, which was associated to allelic loss in 4p in several tumor types, including breast [56], and *FOXP4*, an important member of the forkhead box transcription factors, which are involved in tumorigenesis and cell growth [57]. Although not directly implicated in tumorigenesis, other genes that are part of families involved in cancer appear to be promising candidates. These include *SERPINF2*, a member of the serpin family with a clear role in cancer cell survival [58], *PARD6A,* a member of the PAR family involved in cell cycle gatekeeping and interacting with major cancer pathways like MAPK and PI3K [59], and *HIST2H2AB*, part of the cluster 2 of histones whose parallel family in cluster 1 is highly mutated in many cancer types [60, 61].

#### Gene-wise interactions

We aggregated all the identified variants to check for possible interactions between germline and somatic mutations. We collected 27 variants from known CPGs, 183 variants overlapping with somatic mutations, 41 truncating variants from 38 TSG-like genes and 19 variants from our polygenic model for a total of 254 unique variants in 169 genes. We first checked for a sustainability of the signal of our variants by examining whether a germline variant carrier was more or less prone to have a somatic hit on the same gene (Additional file 1 Gene-wise interactions). Three genes demonstrated a certain propensity to have both somatic and germline hits: *HNF1B*, *MSH6*, *POLR1A* (Additional file 3: Table S9). *HNF1B* has been shown to harbor biallelic inactivation from germline and somatic hits in renal carcinoma [62]. On the 36 *MSH6* carriers of at least one of the three variants we identified, two of them were found carrying also a secondary somatic mutation on the same gene, one of them was a stop-gain truncating mutation. A two-hit hypothesis on *MSH6* has been proposed in [63, 64]. Furthermore, although not significant because of the small sample size, the somatic mutational burden of the patients carrying a *MSH6* variant was slightly higher compared to non-carriers (median for carriers: 33 mutations, median for non-carriers 30 mutations). This is expected from a gene belonging to the mismatch repair pathway. *POLR1A*, which is a core subunit of RNA polymerase 1, has never been shown to necessitate a biallelic inactivation.

#### Subtype-specific variants

Considering the clinical data of our cohort and extracting the estrogen receptor status (ER), the human epidermal growth factor receptor 2 status (HER2), and the progesterone receptor status (PR) for each patient, we tested the possible association between variant carriers and a particular molecular subtype. As a proof of concept, we initially checked if *BRCA1* carriers were associated with ER-negative tumors as previously described [65]. The frequency of ER-negative tumors in non-altered *BRCA1* patients was approximately 20%. Among the *BRCA1* carriers of a pathogenic, highly deleterious or truncating variant (five samples), we found three ER-negative, one positive and one unknown status (3/4 = 75%). By running Fisher’s exact test between *BRCA1* carriers and *BRCA1* non-carriers distributions, we found a significant enrichment of ER-negative tumors (*p* value = 0.025). We expanded this analysis by including the following subtype categories determined by ER, HER2 and PR status: Luminal tumors (ER+/HER2-), HER2+ tumors and triple-negative tumors (ER-, HER2-, PR-). Among the 169 genes taken into consideration in the section above, only two showed a significant imbalance of the distribution of molecular subtypes between carriers and non-carriers (*SMOX*, a spermine oxidase, q-value = 0.02 and *CNOT3*, one of the subunits of CCR4-NOT transcription complex, *p* value = 0.047 - Table S10). For both genes, carriers had more frequently HER2+ tumors compared to non-carriers, (from 2.5% to 25% for *SMOX* and from 2.4% to 9% for *CNOT3*). We did not find any particular connection between these two genes and HER2-positive tumors in the literature, thus we think that our result requires further investigation.

## Discussion

Our study represents one of the first attempts to prioritize germline variants that may predispose to breast cancer using publicly available sequencing data.

We developed a computational framework based on the characteristics of somatic mutations to identify putative cancer-predisposing variants. In particular, we provided an analysis of rare variants, observing that variants in known CPGs are frequent in sporadic TCGA cases. In addition, we detected 183 variants that overlap with somatic mutations in cancer. Furthermore, we carried out an analysis of truncating mutations on suspected tumor suppressors, uncovering both known and novel loss-of-function candidates. We detected 41 variants associated with possible loss-of-function in 38 genes, including *PIK3CB* and *KMT2C*. Lastly, we built a robust age-dependent polygenic model that involves a mixture of supervised and regression-based algorithms to uncover variants at any frequency level. With this model, we identified a set of 19 variants potentially pathogenic and negatively associated with age at onset, and belonging to genes that have never been associated to breast cancer. Finally, we checked if any of the identified candidate variants fell into GWAS known breast cancer susceptibility regions.

We detected several expected variants on known breast cancer-predisposing genes like *BRCA1* and *BRCA2,* which are a confirmation of the validity of this study. We also identified 11 variants on genes known to predispose to other cancer types or cancer syndromes, like *RET* and *AKT1,* which have never been previously associated with breast cancer predisposition.

To our knowledge, there are few examples in the literature that attempt an analysis of predisposing genetic makeups in cancer by exploiting sequencing data [66, 67]. While these works provide an in-depth analysis of known predisposing genes, they lack of a sufficiently extended control dataset; for instance, in the two referenced studies, the authors used, respectively approximately 400 normal controls, against a dataset of ovarian cancer cases of approximately the same size [66], and approximately 1000 samples, against approximately 4000 cases of various cancer types [67]. The use of the ExAC database, which comprises over 27,000 control samples, allowed higher resolution, which we emphasized at the level of the single variants within a candidate predisposing gene, separating variants of scarce significance from true candidate pathogenic variations. Furthermore, in our knowledge-based approach, we introduced more variables, also including over 20 years of breast cancer GWAS data and patients’ characteristics like age at onset. In particular, the latter information was used not only to confirm the association between early onset of disease and known predisposing genes but as a new explanatory variable to further enlarge our set of candidates beyond the limits of already known cancer-related genes.

With our analysis we provided a detailed study of missense variants per se and we offered a way to prioritize cancer-predisposing variants, while previous analysis were more descriptive and mainly focused on truncation events and on loss of heterozygosity. In particular, for breast cancer, previous analysis [67] listed all the rare germline truncation variants present in 624 cancer-associated genes and performed burden test to identify genes with significant enrichment of rare truncations. While they were able to detect *BRCA1* and *BRCA2* as significant in Caucasians, they focused their attention on further characterizing these mutations and on evaluating co-occurrence and mutual exclusivity of *BRCA1*/*BRCA2* germline and somatic variants but there is no attempt at a prioritization of new candidates, especially at the missense variant level.

We are aware that our analysis has several limitations. First, to improve our understanding of the association of rare variants with breast cancer hereditability, we should sequence a larger number of individuals and possibly extend our analysis to other ethnicities. For example, we should use an independent longitudinal cohort to clarify the prevalence of the identified variants, or a smaller cohort of suspected familial cases. Second, genomic data could be associated to patients’ family history, since this information is missing in the TCGA clinical data.

Nevertheless, we have provided a valuable resource of potential new cancer-related variants that can be characterized from a functional point of view.

## Conclusions

In this study, we have developed a genomic-driven approach able to prioritize cancer-predisposing variants using a case-control genetic scheme. We demonstrate the benefits of using publicly available sequencing data to characterize known susceptibility genes, and to identify novel cancer-predisposing variants. The opportunity to classify individuals according to their risk of developing hereditary-based cancer will improve clinical management of breast cancer patients in terms of genome-tailored prevention strategies, programs for early diagnosis, and possible treatments.

## Additional files

Additional file 1: Supplementary methods. (DOCX 64 kb)

Additional file 2: Table S1-5. with reference set of genes and SNPs used in this work. (XLSX 102 kb)

Additional file 3: Table S6-10. with results from SNPs in breast cancer-associated blocks, SNPs overlapping with somatic mutations and tested for truncations in candidate tumor suppressor genes. (XLSX 157 kb)

## Abbreviations

BAM: Binary Alignment/Map
CIViC: Clinic Interpretation of Variants in Cancer
COGS: Collaborative Oncological Gene-environment Study
COSMIC: Catalogue of Somatic Mutations in Cancer
CPGs: Cancer-predisposing genes
DoCM: Database of Curated Mutations
ER: Estrogen receptor
ExAC: Exome Aggregation Consortium
FDR: False discovery rate
GATK: Genome Analysis Toolkit
GWAS: Genome-wide association study
HER2: Human epidermal growth factor receptor 2
Kbac: Kernel-based adaptive cluster test
LD: Linkage disequilibrium
MAF: Minor allele frequency
NFE: Non-Finnish European
NGS: Next-generation sequencing
PR: Progesterone receptor
SKAT-O: Sequence kernel association test optimal
SMGVs: Somatically mutated germline variants
SNP: Single nucleotide polymorphism
SNV: Single nucleotide variant
TCGA: The Cancer Genome Atlas
WES: Whole exome sequencing
WGS: Whole genome sequencing
