# Supplementary material for: A knowledge-based framework for the discovery of cancer-predisposing variants using large-scale sequencing breast cancer data
Source: Breast Cancer Res. 2017 May 31;19:63. doi: 10.1186/s13058-017-0854-1 (PMC5452392; doi:10.1186/s13058-017-0854-1)
Supplement: Supplementary file 1 — Supplementary methods. (DOCX 64 kb) [file 13058_2017_854_MOESM1_ESM.docx]

**Supplementary Methods**

***Study Data***

*Annotation Data*

To better characterize our variants, we took advantage of several external databases and in-house datasets for annotation. In particular, we used ANNOVAR to obtain information on genes, protein changes, and type of variants (missense mutations, truncating mutations, InDels, etc.) [1]. Only variants found within the coding sequence and classified as non-synonymous were retained. ANNOVAR also annotated the variants for “predicted phenotypic effect” with 9 tools included in the dbNSFP database [2] (SIFT [3], Polyphen2_HDIV, Polyphen2_HVAR [4], LRT, MutationTaster [5], MutationAssessor [6], FATHMM [7], RadialSVM, LR [8]). We summarized this information as a comprehensive deleteriousness score (DS), calculated as the proportion of tools calling a particular variant as damaging or probably damaging. Finally, ANNOVAR provided information about the presence of the variant as a somatic mutation in the COSMiC database [9], and of its presence in the ClinVar database [10]. This information was integrated with custom annotations from other public resources. In particular, we annotated whether the variant was present in the cBioPortal database [11] for the same amino acidic change, and in other databases of known disease causing and cancer related mutations, such as CIViC (https://CIViC.genome.wustl.edu), DoCM (http://docm.genome.wustl.edu/), and Humsavar (http://www.uniprot.org/docs/humsavar). These resources provided evidence of some overlap between our case variants and somatic mutations in cancer. The annotated genes were flagged if they belonged to at least one of three categories according to biological and cancer related characteristics: i) genes known to be predisposing for cancer, ii) genes known to be driver in cancer at the somatic level, and iii) genes involved in DNA repair. The first list was created based on the most recent literature, including [12–15] and it represents the state–of-the-art of the knowledge on CPGs (315 genes). The list of known somatic drivers was created using the following 6 tools for detecting driver genes: DOTS-Finder [16], MutSigCV [17], MuSiC [18], OncodriveCLUST [19], OncodriveFM [20], and TUSON [21]. We identified 413 somatic driver genes, considering only the genes predicted to be drivers by at least two different computational methods. Finally, we added a comprehensive list of 166 genes involved in DNA repair [22]. In total, we considered 758 genes as our target gene list (**Additional File 1 – Table S1**). In addition, we also classified these genes as potential oncogenes or tumor suppressors, based on the joint results from literature annotations and from those tools that classify genes in tumor suppressors or oncogenes based on their mutational patterns [12,13,16,21,23]. In case of discordant results, the gene was considered both a tumor suppressor and an oncogene. In total, we classified 119 genes as oncogenes and 235 genes as tumor suppressors (**Additional File 1 – Table S2**). We retrieved a dataset of known breast cancer associated SNPs from the GWAS studies included in the Human Genome Research Institute’s Catalog of Published Genome-wide Association Studies (NHGRI-EBI GWAS Catalog version 2016-05-08) [24]. Each SNP was reported with a p-value based on the manual curation of the literature about treat/disease association SNPs (TASs) as reported in Hindorff *et al.* [25]. We manually selected the publications included in the catalog under the ontology “breast cancer” with studies in cohorts of European origin during the discovery phase and presenting SNPs associated with the disease and not with some of its characteristics with at least a p-value of 5e10^-8^, which is the default value used in the catalog. For example, we excluded studies about drug resistance, chemotherapy adverse events, or levels of proteins in breast tissues. These variants are not directly associated with the disease but represent a flag for a probable region where the disease-causing variant might be found. In total, we collected a list of 130 unique SNPs from 23 studies (see **Additional File 1 – Table S3**). The table contains 177 hits because some of the SNPs are confirmed by more than one study. Using HapMap recombination data [26], we created the boundaries of such regions, defined as all the DNA regions surrounding the GWAS SNPs below a recombination rate of 20 cM/Mb (linkage disequilibrium blocks, LD blocks) [27]. If one of our variants fell into one of these regions, its distance from the flag GWAS SNP was annotated (**Additional File 1 – Table S4**). Actually, there is no direct relationship between physical distance and genetic distance but, since we are inside low recombination regions, we can consider base pair distance as a proxy for cM distance.

*Data Preprocess*

Case data preprocess was based on the whole GATK pipeline used by the ExAC consortium to avoid pipeline specific biases [28]. This included Picard MarkDuplicates, local realignment around InDels, base quality recalibration, haplotype call, joint genotyping, and variant quality score recalibration (http://broadinstitute.github.io/picard) [29]. Our pipeline also included the splitting of multiallelic sites and left alignment, in order to phase both cases and controls more easily. In order to obtain robust genotype calls, we retained genotype calls with genotype quality higher than 20 and depth of sequencing higher than 10.

As breast cancer is much more common in women than men, our case dataset was composed of 7 men and 666 women. We therefore fixed the ploidity for men on chromosome X in non-pseudo autosomal regions. We also checked that all the variants called were genuine germline alterations by comparing them with the somatic mutations calls available from the TCGA for all our 673 cases. We used bcftools/vcftools, variant tools (vt) [30,31] and in-house scripts to process the post-call data.

***Statistical Analyses***

*Annotation based analysis*

The annotation based analysis (used in the first two sections of the results) is made up of a simple annotation and filtering step-wise procedure (as summarized in **Fig. 3**). Rare non-synonymous variants were retained, and only those with a case MAF greater than the controls kept (control MAF below 1%). Subsequently, only damaging mutations with a DS of at least 0.5 (*i.e.,* the majority of tools for prediction of phenotypic effect considering the mutation as damaging) were selected. The pipeline was then divided in two branches. On the one side (right arm of **Fig. 3**), we looked for those variants that had been classified as somatic in any type of tumor using the COSMIC and the cBioPortal databases. On the other side, we took into consideration only variants that fell into the LD blocks of previously annotated breast cancer associated SNPs in GWAS studies (see section *Method – Study data - Annotation Data*).

*Tumor suppressor-like analysis*

In this analysis, we performed a gene-wise test that looks for imbalance in the allele counts in truncation events between cases and controls (Loss-of-function testing, LOF). In this testing procedure, we looked for truncating variants, *i.e.*, frame shift InDels or nonsense mutations that retain, by definition, a higher probability of creating a loss-of-function event. In this context, we wanted to replicate the method by which driver somatic tumor suppressor genes are generally discovered, using the frequency of any rare truncation compared to the same frequency in the control cohort [13,16,21]. We first filtered out common events (over 1% in the control cohort) and then we performed, for every variant, a simple Fisher’s exact test between minor/major allele counts between cases and controls. For every gene, we aggregated all the p-values obtained from the tests using the Stouffer method [32] to obtain a single value per gene. In this procedure, a weighting proportional to the inverse of the control frequency was applied so that the rarer the variant, the higher the weighting applied in the aggregation step. Finally, amongst the genes belonging to our target gene list, we retained only those with an FDR corrected p-value below 0.05.

*Age-dependent polygenic modeling*

The Age-dependent polygenic model branch is a step-wise procedure. Like for the LOF procedure, we calculated a minor/major variant-wise Fisher’s exact test between cases and controls, including all variants, without applying any filter to the MAF of the control cohort. For multiallelic sites, references and alternatives composed a matrix of 2 rows (controls and cases) by *n+1* columns, where 1 represents the reference counts and n the number of different alternative alleles. A bootstrap version of Fisher’s test was used in this case. The calculated p-values were added as an explanatory variable in the step-wise procedure. We also ran other commonly used human genetic statistical tests and the p-values obtained were added as explanatory variables, both gene-wise and variant-wise. Using RVtest [33], we were able to run SKAT-O [34], CMC [35], and Kbac [36] tests for the gene-wise level, and Wald and SingleScore [37] tests for the variant-wise level. All the aforementioned tests used the 1000 genome original genotype calls [38] (for the Non Finnish European cohort) as the control cohort, since we needed the full genotypes in order to run them and they were not available for the ExAC database. These statistical tests are not used as such but represent features of a classification problem described hereby.

The workflow:

1) We selected a set of variants from our case samples that account for every variation reported as pathogenic in at least one of the following databases: Humsavar, DoCM, ClinVar or CIViC. Variants were further subset for a manually curated list of cancer related keywords. This list included predisposition to both straight cancer and neoplastic events, as well as to cancer-related syndromes (like Li-Fraumeni or Von Hippel Lindau syndromes), for a total of 38 variants in 24 genes (**Additional File 1 – Table S5**).

2) We selected a set of negative control variants from the list of ClinVar annotated variants that were established as non-pathogenic, for a total of 706 variants.

3) We implemented a random forest classifier with 100,001 trees, using a dichotomous response variable (pathogenic, non-pathogenic) with a training set that included all the variants in point 1) and 2) [39]. The features used for classification are reported in **Fig. 4a** and include all the tests described above, MAF in cases and controls, number of homozygous and heterozygous calls, DS, and a dummy variable describing the type of variant (a truncation event or a simple missense variant). A tree-based algorithm like the random forest is particularly powerful in situations where the interaction of various features is critical for the model. The output of this analysis was the relative number of trees classifying a variant as pathogenic (a proportion between 0 and 1). We named this score random forest (RF) score.

4) We selected the variants that did not belong to the training set and filtered for those showing a RF score of at least 0.5 (majority voting in the random forest procedure)

5) We correlated our variants with age at initial pathological diagnosis. The variants found at points 3-4) switched from being subjects to becoming explanatory variables, with a value of 0, 1 or 2 according to the state of the homozygous major allele and of the heterozygous or homozygous minor alleles. With such a dataset, we built a robust elastic net linear model by running, in parallel, 100 models under various random subsets of the dataset, where each feature was weighted in the model by the RF score obtained at point 4) [40]. This procedure guarantees that the average beta values and the number of times a feature is in all the elastic net models remain stable. A penalized linear model like the elastic net is preferable for its ability of assessing the direction and the magnitude of the contribution of each variant. In the case of age for example, we were interested in detecting the variants with a negative beta, in other words, those that contributed to decreasing the age at onset. Considering that a male has one hundred times less risk of getting cancer than a female of the same age, a male patient age was rescaled with a logit function in order to correspond to the same risk of a younger woman. To build the risk function, we used data from the Cancer Research UK report 1996-2011, available at <http://www.cancerresearchuk.org/health-professional/cancer-statistics/statistics-by-cancer-type/breast-cancer>.

*Gene-wise interactions*

To assess whether samples that harbor variants on a gene are more or less prone to develop a secondary somatic mutation on the same gene we developed a permutation test that extracted a number of random patients equal to the number of carriers for 10’000 times from the pool of non-carriers. We then assessed how many times our somatic mutation burden was equal or superior or equal or inferior to the one obtained by random sampling. For this analysis we only considered genes with at least two or more carriers.

**References**

1. Wang K, Li M, Hakonarson H. ANNOVAR: functional annotation of genetic variants from high-throughput sequencing data. Nucleic Acids Res. 2010;38:e164.

2. Liu X, Jian X, Boerwinkle E. dbNSFP v2.0: A Database of Human Non-synonymous SNVs and Their Functional Predictions and Annotations. Hum. Mutat. 2013;34:E2393–402.

3. Sim N-L, Kumar P, Hu J, Henikoff S, Schneider G, Ng PC. SIFT web server: predicting effects of amino acid substitutions on proteins. Nucleic Acids Res. 2012;40:W452–7.

4. Adzhubei I, Jordan DM, Sunyaev SR. Predicting Functional Effect of Human Missense Mutations Using PolyPhen-2. Curr. Protoc. Hum. Genet. Editor. Board Jonathan Haines Al. 2013;0 7:Unit7.20.

5. Schwarz JM, Rödelsperger C, Schuelke M, Seelow D. MutationTaster evaluates disease-causing potential of sequence alterations. Nat. Methods. 2010;7:575–6.

6. Reva B, Antipin Y, Sander C. Predicting the functional impact of protein mutations: application to cancer genomics. Nucleic Acids Res. 2011;39:e118–e118.

7. Shihab H a., Gough J, Cooper DN, Stenson PD, Barker GL a, Edwards KJ, et al. Predicting the Functional, Molecular, and Phenotypic Consequences of Amino Acid Substitutions using Hidden Markov Models. Hum. Mutat. 2013;34:57–65.

8. Li Q, Liu X, Gibbs RA, Boerwinkle E, Polychronakos C, Qu H-Q. Gene-Specific Function Prediction for Non-Synonymous Mutations in Monogenic Diabetes Genes. PLOS ONE. 2014;9:e104452.

9. Forbes SA, Bhamra G, Bamford S, Dawson E, Kok C, Clements J, et al. The Catalogue of Somatic Mutations in Cancer (COSMIC). Curr. Protoc. Hum. Genet. Editor. Board Jonathan Haines Al. 2008;CHAPTER:Unit-10.11.

10. Landrum MJ, Lee JM, Riley GR, Jang W, Rubinstein WS, Church DM, et al. ClinVar: public archive of relationships among sequence variation and human phenotype. Nucleic Acids Res. 2014;42:D980–5.

11. Cerami E, Gao J, Dogrusoz U, Gross BE, Sumer SO, Aksoy BA, et al. The cBio Cancer Genomics Portal: An Open Platform for Exploring Multidimensional Cancer Genomics Data. Cancer Discov. 2012;2:401–4.

12. Rahman N. Realizing the promise of cancer predisposition genes. Nature. 2014;505:302–8.

13. Vogelstein B, Papadopoulos N, Velculescu VE, Zhou S, Diaz LA, Kinzler KW. Cancer Genome Landscapes. Science. 2013;339:1546–58.

14. Walsh T, Lee MK, Casadei S, Thornton AM, Stray SM, Pennil C, et al. Detection of inherited mutations for breast and ovarian cancer using genomic capture and massively parallel sequencing. Proc. Natl. Acad. Sci. 2010;107:12629–33.

15. Futreal PA, Coin L, Marshall M, Down T, Hubbard T, Wooster R, et al. A census of human cancer genes. Nat. Rev. Cancer. 2004;4:177–183.

16. Melloni GE, Ogier AG, de Pretis S, Mazzarella L, Pelizzola M, Pelicci PG, et al. DOTS-Finder: a comprehensive tool for assessing driver genes in cancer genomes. Genome Med. 2014;6:44.

17. Lawrence MS, Stojanov P, Mermel CH, Robinson JT, Garraway LA, Golub TR, et al. Discovery and saturation analysis of cancer genes across 21 tumour types. Nature. 2014;505:495–501.

18. Kandoth C, McLellan MD, Vandin F, Ye K, Niu B, Lu C, et al. Mutational landscape and significance across 12 major cancer types. Nature. 2013;502:333–9.

19. Tamborero D, Gonzalez-Perez A, Lopez-Bigas N. OncodriveCLUST: exploiting the positional clustering of somatic mutations to identify cancer genes. Bioinformatics. 2013;29:2238–44.

20. Gonzalez-Perez A, Lopez-Bigas N. Functional impact bias reveals cancer drivers. Nucleic Acids Res. 2012;40:e169.

21. Davoli T, Xu AW, Mengwasser KE, Sack LM, Yoon JC, Park PJ, et al. Cumulative Haploinsufficiency and Triplosensitivity Drive Aneuploidy Patterns and Shape the Cancer Genome. Cell. 2013;155:948–62.

22. Lange SS, Takata K, Wood RD. DNA polymerases and cancer. Nat. Rev. Cancer. 2011;11:96–110.

23. Schroeder MP, Rubio-Perez C, Tamborero D, Gonzalez-Perez A, Lopez-Bigas N. OncodriveROLE classifies cancer driver genes in loss of function and activating mode of action. Bioinformatics. 2014;30:i549–55.

24. Welter D, MacArthur J, Morales J, Burdett T, Hall P, Junkins H, et al. The NHGRI GWAS Catalog, a curated resource of SNP-trait associations. Nucleic Acids Res. 2014;42:D1001–6.

25. Hindorff LA, Sethupathy P, Junkins HA, Ramos EM, Mehta JP, Collins FS, et al. Potential etiologic and functional implications of genome-wide association loci for human diseases and traits. Proc. Natl. Acad. Sci. 2009;106:9362–7.

26. Frazer KA, Ballinger DG, Cox DR, Hinds DA, Stuve LL, Gibbs RA, et al. A second generation human haplotype map of over 3.1 million SNPs. Nature. 2007;449:851–61.

27. Machiela MJ, Ho BM, Fisher VA, Hua X, Chanock SJ. Limited evidence that cancer susceptibility regions are preferential targets for somatic mutation. Genome Biol. 2015;16:193.

28. Lek M, Karczewski KJ, Minikel EV, Samocha KE, Banks E, Fennell T, et al. Analysis of protein-coding genetic variation in 60,706 humans. Nature. 2016;536:285–91.

29. McKenna A, Hanna M, Banks E, Sivachenko A, Cibulskis K, Kernytsky A, et al. The Genome Analysis Toolkit: A MapReduce framework for analyzing next-generation DNA sequencing data. Genome Res. 2010;20:1297–303.

30. Danecek P, Auton A, Abecasis G, Albers CA, Banks E, DePristo MA, et al. The variant call format and VCFtools. Bioinformatics. 2011;27:2156–8.

31. Tan A, Abecasis GR, Kang HM. Unified representation of genetic variants. Bioinformatics. 2015;31:2202–4.

32. Stouffer S, DeVinney L, Suchmen E. The American soldier: Adjustment during army life. Princeton University Press. Princeton US: Princeton University Press; 1949.

33. Zhan X, Hu Y, Li B, Abecasis GR, Liu DJ. RVTESTS: an efficient and comprehensive tool for rare variant association analysis using sequence data. Bioinformatics. 2016;32:1423–6.

34. Lee S, Emond MJ, Bamshad MJ, Barnes KC, Rieder MJ, Nickerson DA, et al. Optimal Unified Approach for Rare-Variant Association Testing with Application to Small-Sample Case-Control Whole-Exome Sequencing Studies. Am. J. Hum. Genet. 2012;91:224–37.

35. Li B, Leal SM. Methods for Detecting Associations with Rare Variants for Common Diseases: Application to Analysis of Sequence Data. Am. J. Hum. Genet. 2008;83:311–21.

36. Liu DJ, Leal SM. A Novel Adaptive Method for the Analysis of Next-Generation Sequencing Data to Detect Complex Trait Associations with Rare Variants Due to Gene Main Effects and Interactions. PLOS Genet. 2010;6:e1001156.

37. Xing G, Lin C-Y, Wooding SP, Xing C. Blindly Using Wald’s Test Can Miss Rare Disease-Causal Variants in Case-Control Association Studies. Ann. Hum. Genet. 2012;76:168–77.

38. 1000 Genomes Project Consortium, Abecasis GR, Auton A, Brooks LD, DePristo MA, Durbin RM, et al. An integrated map of genetic variation from 1,092 human genomes. Nature. 2012;491:56–65.

39. Breiman L. Random forests. Mach. Learn. 2001;45:5–32.

40. Zou H, Hastie T. Regularization and variable selection via the elastic net. J. R. Stat. Soc. Ser. B Stat. Methodol. 2005;67:301–320.
